# Exploring the Potential of Low-Temperature Vacuum Drying to Improve the Bioactive Compound Content and Health-Promoting Properties of Chilean Wild Murta

**DOI:** 10.3390/antiox14101201

**Published:** 2025-10-03

**Authors:** Antonio Vega-Galvez, Alexis Pasten, Elsa Uribe, Nicol Mejias, Isadora Corco, Jacqueline Poblete, Jaime Ortiz-Viedma, Gabriela Valenzuela-Barra, Javier Acevedo-Hernández, Tamar Toledo

**Affiliations:** 1 Food Engineering Department, Universidad de La Serena, Av. Raúl Bitrán 1305, La Serena 1700000, Chile; afpasten@userena.cl (A.P.); muribe@userena.cl (E.U.); susana.mejias@userena.cl (N.M.); isadorapaz.corco@userena.cl (I.C.); j.pobletegalleguillos@gmail.com (J.P.); 2 Departamento de Ciencias de los Alimentos y Tecnología Química, Facultad de Ciencias Químicas y Farmacéuticas, Universidad de Chile, Av. Dr. Carlos Lorca Tobar 964, Independencia, Santiago 8330111, Chile; jaortiz@uchile.cl; 3 Laboratorio de Productos Naturales, Facultad de Ciencias Químicas y Farmacéuticas, Universidad de Chile, Santiago 8380000, Chile; gabriela.m.valenzuela@ciq.uchile.cl (G.V.-B.); javier.acevedo.h@ug.uchile.cl (J.A.-H.); 4 Department of Analytical Chemistry, Nutrition and Food Science, Food Technology Division, School of Veterinary Sciences, Universidad de Santiago de Compostela, 15782 Lugo, Spain; tamar.toledoaquino@gmail.com

**Keywords:** low-temperature vacuum drying, native wild berries, phlogistic agents

## Abstract

For the first time, the effect of low-temperature vacuum drying (LTVD) on wild murta (*Ugni molinae* Turcz) was evaluated, in comparison with freeze-drying (FD) and vacuum drying (VD), to assess their capacity to preserve bioactive compounds and associated bioactivities. Murta was dried using LTVD at 20, 30, and 40 °C under a constant vacuum of 10 mbar, where FD and VD at 60 °C (VD 60) were included as comparative methods. The content of fatty acids and tocols, along with the retention of bioactive compounds and their antioxidant, anti-inflammatory, cytotoxic, and α-glucosidase inhibitory activities, were systematically analyzed. LTVD- and VD-dried murta exhibited higher polyunsaturated-to-saturated fatty acid ratios (>9.0) and markedly greater tocol contents, whereas FD maintained a more balanced ratio (<5.0) but with lower tocol levels. While FD was most effective in preserving catechin, higher levels of other phenolic compounds were observed in samples dried by LTVD at 20 and 40 °C, as well as VD 60, possibly due to the release of bound forms during processing. The drying method significantly influenced murta bioactivity. LTVD 30 preserved the highest antioxidant capacity, while topical anti-inflammatory effects on skin lesions varied by pathway, with LTVD 40 being the most effective in the TPA model and FD in the AA model. These effects were evaluated only using a topical inflammation model in BALB/c mice of both sexes; dietary effects were not assessed in this study. Regarding other bioactivities, VD 60 extracts excelled in both cytotoxic and α-glucosidase inhibitory effects, whereas FD extracts were the most effective against AGS cells and LTVD 20 against α-glucosidase. In conclusion, LTVD emerges as a promising alternative to FD and VD, showing potential to preserve bioactive compounds and key bioactivities of wild murta, although further studies are needed to elucidate the underlying mechanisms.

## 1. Introduction

Chile is considered one of the world’s five major biodiversity hotspots, hosting several endemic berry species. Among them, murta (*Ugni molinae* Turcz.) is a small evergreen shrub in the Myrtaceae family, native to south-central Chile [1,2,3]. It produces pink, spherical berries (0.71–1.31 cm in diameter) valued for their sweet aroma, pleasant flavor, and nutritional properties [4,5].

Its use as both food and medicine by indigenous communities has prompted growing scientific interest in elucidating the mechanisms underlying its effects on certain health conditions. In line with this interest, studies have identified more than 100 bioactive compounds in murta, including simple phenols, phenolic acids, flavonols, flavones, and proanthocyanidins [4,6,7,8,9,10,11,12,13], as well as pentacyclic triterpenoids in the leaves [14,15,16] and different, along with distinct tannin fractions in the seeds [9]. These bioactive compounds may account for the astringent, anti-inflammatory, digestive, and regenerative effects attributed to murta in the Mapuche medical system, where it has been traditionally used in infusions, fermented beverages, and topical applications within a therapeutic framework that combines empirical practices and ancestral shamanism [2,3,17].

Beyond its traditional medicinal uses, the majority of murta is consumed fresh, although part of the production is processed by the food industry into dried fruits, juice, jam, canned products, and liqueurs to ensure its availability throughout the year [1,5,9]. Moreover, roasted seeds can serve as a coffee substitute and, similar to dried fruits, can be incorporated into herbal infusions or various food preparations [3]. However, conventional processing techniques, which generally involve heat treatments, can degrade the bioactive compounds in murta, underscoring the need for novel low-temperature processing strategies.

In fact, for a high-value commodity such as murta berries, which command premium market prices, the use of freeze-drying (FD) is justified despite being an expensive drying technique. Nevertheless, alternative methods such as low-temperature vacuum drying (LTVD) have emerged as more energy-efficient options while still preserving product quality. This innovative technique combines reduced pressure and lower drying temperatures compared to conventional vacuum drying (VD), and offers better energy efficiency than FD by eliminating the freezing step followed by sublimation [18,19]. LTVD operates on a principle similar to vacuum cooling, in which lowering the ambient pressure reduces the boiling point of water, allowing evaporation to occur at lower temperatures. However, unlike vacuum cooling, which is designed primarily for rapid temperature reduction, LTVD applies this principle to achieve gradual dehydration while preserving bioactive compounds [20]. This innovative approach not only enhances the overall quality of food products but also produces dried products with colors and flavors comparable to those obtained through FD [21]. By enabling substantial reductions in drying temperature, LTVD contributes to the production of minimally processed foods.

Even though the effects of different drying methods on murta have been previously reported [7,22,23], to date, no studies have evaluated murta dried using LTVD. Conventional VD operates at higher temperatures, which may compromise the stability of thermolabile compounds, while FD (although highly effective in preserving quality) is limited by its high cost, long processing times, and high energy demand. Therefore, the aim of the present study was to evaluate the effect of LTVD at different temperatures (20, 30, and 40 °C) under a constant vacuum pressure of 10 mbar on *Ugni molinae* Turcz. This assessment encompassed fatty acid and tocol contents, along with the retention of bioactive compounds and their associated antioxidant, anti-inflammatory, cytotoxic, and α-glucosidase inhibitory activities. Moreover, the results were compared with those obtained using conventional VD and FD.

## 2. Materials and Methods

### 2.1. Sample Preparation for Vacuum-Based Drying Processes

Wild murta berries (*Ugni molinae* Turcz) were obtained from a commercial supplier (AIMA Chile, Osorno, Chile) to ensure compliance with quality standards of freshness, wholesomeness, color, and uniformity. Upon reception, the berries were stored at 4 °C for 24 h. Since pretreatment was required to enhance moisture diffusion during low-temperature drying, the whole fruits were subjected to an enzymatic step with 1% (*v*/*v*) pectinase (Rohapect 10-L, Dimerco Commercial S.A., Santiago, Chile). The enzyme solution was prepared in a 50 mM citrate buffer adjusted to pH 4.5. Whole murta berries were immersed in the enzyme solution at a ratio of 1:3 (*w*/*v*) and maintained under continuous stirring in a water bath (N-Biotek, NB-302, Bucheon-si, Gyeonggi-do, Republic of Korea) at 50 °C for 30 min. After this step, the fresh murta berries were thoroughly rinsed and allowed to drain to remove residual surface moisture. The enzymatic pretreatment was applied consistently to all samples prior to drying, regardless of the method used, to ensure comparability. It should be noted that this pretreatment was applied with the specific purpose of promoting a partial degradation of pectin at the fruit surface, thereby facilitating moisture diffusion through the peel and accelerating the drying process. It was not intended as a factor for evaluating changes in bioactive components.

### 2.2. Vacuum-Based Drying Processes

The drying of murta was carried out using a low-temperature vacuum drying (LTVD) system designed for laboratory applications (Memmert VOcool 400, Schwabach, Germany). This equipment is fitted with a compact Peltier cooling unit, which operates at temperatures below 20 °C, and an aluminum thermoshelf with a working range of 5–90 °C. It also incorporates a digital electronic pressure controller, allowing precise adjustment of vacuum pressure between 5 and 1100 mbar. In this study, pretreated murta samples were evenly distributed in thin layers on the fixed thermoshelf inside the drying chamber. Drying was conducted at 20, 30, and 40 °C, each under a constant vacuum of 10 mbar. Operating the LTVD system at 10 mbar ensured that the process was carried out above the triple point of water (0.01 °C and 6.11 mbar), thus avoiding sublimation and confirming that the technique corresponded to LTVD rather than freeze-drying (FD).

As LTVD represents an intermediate approach between FD and conventional vacuum drying (VD), both techniques were included as comparative methods. For FD, pretreated murta was first frozen at −80 °C and then freeze-dried in a vacuum freeze dryer (Friologic Given One 5K, Santiago, Chile) at a condenser temperature of −55 ± 2 °C under a pressure of 0.267 mbar. For VD, pretreated murta was dried in a vacuum dryer (Memmert VO 400, Schwabach, Germany) coupled to a vacuum pump (Buchi V-100, Flawil, Switzerland) with an ultimate pressure of 100 mbar.

**Figure 1:**
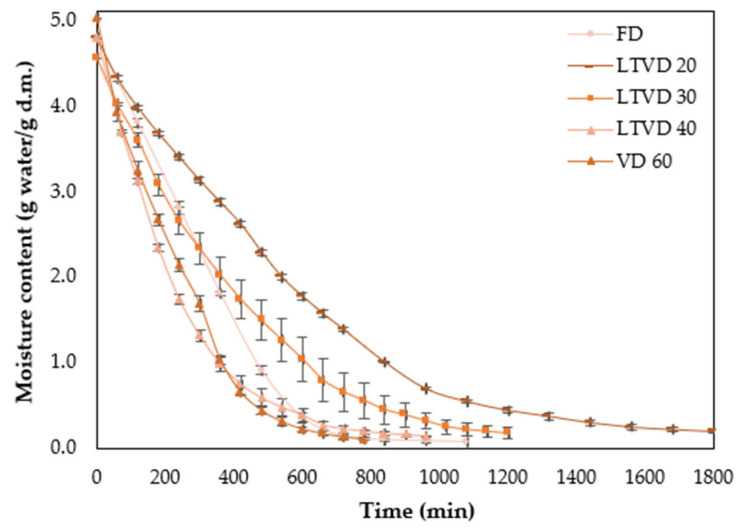
Moisture content (g water/g d.m.) plotted against drying time (min) for murta berries dried by vacuum-based methods (FD, LTVD, and VD). Data are presented as mean values of triplicate analyses (*n* = 3), with error bars representing standard deviations.

The drying time for each method was estimated by monitoring the weight loss of pretreated murta berries (Figure 1). Approximately 30 g of sample was placed in baskets measuring 14 cm in length and 8 cm in width. The initial weight was recorded, and subsequent weights were measured at predetermined time intervals for each drying method using a digital balance SP402 (Ohaus Corporation, Parsippany, NJ, USA.). Drying was continued until equilibrium was reached, as indicated by a constant sample weight. All experiments were conducted in triplicate.

Moisture content during drying was determined using a mass balance equation, assuming that the dry matter remained constant and that the decrease in sample weight was exclusively due to water loss. When expressed on a dry basis (g water/g dry matter, d.m.), moisture content was calculated as follows:

(1) $$X_{t}=\frac{W_{t}-W_{s}}{W_{s}}$$

where $W_{t}$ is the sample weight at time *t*, and $W_{s}$ is the dry solid weight determined after drying to constant weight according to the AOAC 934.06 method.

Upon completion of the drying process, samples were ground to a homogeneous powder and stored in high-density polyethylene bags under refrigeration and protected from light. Additionally, both fresh and dried murta samples were characterized through routine proximate analysis, including moisture content determined according to the AOAC 934.06 procedure. The results are provided in the Supplementary Material (Table S1) as supporting information only.

### 2.3. Fatty Acids

The fatty acids profile was determined by gas chromatography according to the AOCS method Ce 2-66 A [24] using an GC-2014 (Shimadzu Corp., Tokyo, Japan) with a FID detector, auto injector AOC-20i (Shimadzu Corp., Tokyo, Japan), using a SP-2560 fused silica capillary column (100 m, 0.25 mm, 0.2 µm film thickness; Supelco, Bellefonte, PA, USA). The temperature was programmed between 160 °C and 230 °C at 2 °C/min. Additionally, 0.5 µL samples were run with nitrogen as the carrier gas. The fatty acid methyl esters (FAME) was identified by retention time relative to the standards (Reference Standard GLC 463, Nu-Chek Prep, Inc., Elysian, MN, USA). Results are expressed as the relative percentage of the fatty acids detected under these analytical conditions, and not as the absolute total fatty acid content.

### 2.4. Tocols

Tocopherols and tocotrienols were determined by high-performance liquid chromatography (HPLC) according to the standard method AOCS Ce 8-89 [25], using a LiChro-CART Superspher Si 60 column (Merck, Darmstadt, Germany). The mobile phase was propan-2-ol in hexane (0.5/99.5 *v*/*v*) and injected at a flow rate of 1 mL/min. The HPLC system consisted of a Waters 1525 binary HPLC pump (Waters Corp., Milford, MA, USA), with the FlexInject Manual Dual Injector Module (Hitachi, Tokyo, Japan), the Chro-master 5440 fluorescence detector (Hitachi, Tokyo, Japan), and the D-2500 Chromato-integrator (Merck-Hitachi, Tokyo, Japan). Peaks were detected at excitation and emission wavelengths of 290 nm and 330 nm, respectively. Tocols were determined using tocopherol and tocotrienol standards (Calbiochem Merck, Darmstadt, Germany). The results were measured in duplicate and expressed in mg TE/100 g oil.

### 2.5. Extraction Procedure

Phenolic compounds were extracted according to a previously reported procedure [22], with minor adjustments. In brief, 2.5 g of dried sample were mixed with 20 mL of 69% acetone and shaken at 300 rpm for 63 min. The extracts were centrifuged at 4193× *g* for 5 min. The extraction was repeated once more with acetone, and the resulting supernatants were pooled and evaporated under reduced pressure using a Multivapor (Büchi P-6, Flawil, Switzerland) at 40 °C. The concentrated extract was subsequently lyophilized to obtain a solid residue, which was stored at −80 °C until analysis.

### 2.6. Identification and Quantification of Individual Phenolic Compounds

The lyophilized murta extracts (solid residue) were reconstituted to 10 mL with methanol–formic acid (99:1, *v*/*v*) prior to chromatographic analysis. Chromatographic separation was performed using an HPLC system (Agilent 1200 Series, Santa Clara, CA, USA) equipped with a diode-array detector (DAD). Prior to injection, extracts were filtered through 0.45 μm membranes, and 10 μL were injected into a Kromasil 100-5C18 column (250 × 4.6 mm, 5 μm; Eka Chemical, Bohus, Sweden). The mobile phase consisted of solvent A (0.1% formic acid, pH 2.6) and solvent B (acetonitrile), with the following gradient program: 87% solvent A and 13% solvent B, followed by a linear change to 45% A and 55% B over 0–16 min. Between 16 and 23 min, the composition shifted to 60% A and 40% B. From 23 to 25 min, the system returned to 87% A and 13% B, which was then maintained until 30 min to re-equilibrate the column. Runs were carried out at 25 °C with a flow rate of 0.7 mL/min. Spectral data were recorded at 280, 310, and 370 nm. A representative chromatogram recorded at 280 nm is provided in the Supplementary Material (Figure S1).

### 2.7. Antioxidant and Anti-Inflammatory Assays

Twenty milligrams of lyophilized murta extract (solid residue) were reconstituted in 1 mL of phosphate buffer (75 mM, pH 7.4) prior to Oxygen Radical Absorbance Capacity (ORAC) analysis. An aliquot of 40 μL of the diluted extract was mixed with fluorescein sodium salt solution (200 μL, 100 nM) in black 96-well plates and incubated at 37 °C for 20 min, after which AAPH solution (35 μL, 0.36 M) was added. Fluorescence intensity was monitored at 37 °C using a microplate reader (excitation: 485 nm; emission: 535 nm) at 1 min intervals until signal decay. A standard calibration curve was generated with Trolox (5–250 μM; y = 0.00002x − 26.664; R^2^ = 0.9908). Results were expressed as μmol Trolox equivalents (TE) per gram of dry matter (d.m.).

All in vivo procedures were conducted in compliance with the NIH Guide for the Care and Use of Laboratory Animals and the AVMA 2020 euthanasia guidelines, and were approved by the Institutional Animal Care and Use Committee (CICUA-VID, University of Chile; protocol 25887-ODO-UCH, approved on 9 April 2025). Juvenile BALB/c mice (20–25 g; either sex) were used in an acute, non-survival design on the same day of dosing. Animals (≤4/cage) had food/water ad libitum. Mice were randomly assigned (simple randomization); the experimental unit was one mouse. Operators who weighed punches and the data analyst were blinded to allocation.

Edema was induced on the right ear with arachidonic acid (AA, 2 mg in 20 µL acetone/ear) or phorbol 12-myristate 13-acetate (TPA, 5 µg in 20 µL acetone/ear). The left ear received acetone (vehicle). Murta (*Ugni molinae*) extracts (3.0 mg/ear in 20 µL ethanol) were applied immediately after the stimulus. Positive controls: nimesulide (0.5 mg/ear, AA model) and indomethacin (1.0 mg/ear, TPA model). Group sizes: stimulus-only control *n* = 16; each treatment and positive control *n* = 8.

At 1 h (AA) or 6 h (TPA) post-induction, mice were euthanized by CO_2_ (gradual-fill), and 6 mm ear punches were collected bilaterally. Edema (mg) was defined as the difference between the medians of the punch weights from the right (inflamed) and left (vehicle) ears for each group; percent inhibition was computed relative to the median of the stimulus-only group. Normality (Shapiro–Wilk); Kruskal–Wallis + Mann–Whitney (α = 0.05) [14].

### 2.8. α-Glucosidase Inhibitory Activity Assay

Forty milligrams of lyophilized murta extract (solid residue) were reconstituted in 1 mL of methanol/water (80:20, *v*/*v*) prior to α-glucosidase inhibitory activity analysis. Briefly, α-glucosidase from *Saccharomyces cerevisiae* was prepared in 0.1 M phosphate buffer, and 4-nitrophenyl-α-D-glucopyranoside (pNPG) was used as the substrate. Aliquots of 50 μL of the reconstituted extract at various concentrations (0.10–40.0 mg/mL) were combined with 100 μL of enzyme solution (0.5 U/mL) and incubated at 25 °C for 10 min. The reaction was initiated by adding 50 μL of pNPG (5 mM), and absorbance was monitored at 405 nm using a microplate reader at 30 s intervals for 10 min at 25 °C. Phosphate buffer served as the blank, whereas acarbose was included as a positive control. The inhibitory effect (%) of murta extracts or acarbose on α-glucosidase activity was calculated using the following equation:

(2) $$\%\;inhibition=\left(\left[\frac{{∆A}_{405}^{blank}-{∆A}_{405}^{inhibitor}}{{∆A}_{405}^{blank}}\right]\right)\times 100$$

where ${∆A}_{405}^{blank}$ represent the absorbance of the blank at the beginning and end of incubation, whereas ${∆A}_{405}^{inhibitor}$ represent the absorbance of the inhibitor at the beginning and end of incubation. The substrate was included in all groups. Nonlinear regression analysis was applied to determine the half-maximal inhibitory concentration (IC_50_).

### 2.9. Cytotoxicity Assay

The human gastric cancer cell line (AGS) used in this study was kindly provided by the Department of Food Engineering, University of Valdivia (Valdivia, Chile). Cells were cultured in F-12K medium (Kaighn’s modification of Ham’s F-12 medium) supplemented with 10% fetal bovine serum (FBS) and 1% antibiotic–antimycotic solution. Cultures were maintained in a CO_2_ incubator (Memmert, INCO153med, Schwabach, Germany) at 37 °C under 5% CO_2_. Only healthy cells that had undergone several passages and reached optimal growth were selected for subsequent experiments.

For cytotoxicity assays, cells (5000 cells/well) were seeded into 96-well plates and incubated for 24 h to reach approximately 80% confluence prior to treatment. Murta extracts, reconstituted in F-12K medium, were applied at concentrations ranging from 1.25 to 20.00 mg/mL to assess the minimum inhibitory concentration against AGS cells. After 24 h of exposure, 100 μL of HBSS-Ca^2+^ solution (40 μM KH_2_PO_4_; 30 μM NaH_2_PO_4_·H_2_O; 13.6 μM NaCl; 600 μM D-glucose; 500 μM KCl; and 900 μM CaCl_2_) containing propidium iodide (PI) was added directly to the existing 100 μL of culture medium, yielding a final PI concentration of 5 μM. Cells were then incubated for 10 min. Untreated cultures in F-12K medium served as the negative control, whereas 50% dimethyl sulfoxide (DMSO) was used as the positive control. Fluorescence was recorded using a microplate reader (TECAN Infinite M Nano+, Maennedorf, Switzerland) at 617 nm and 535 nm. Nonlinear regression analysis was employed to calculate the half-maximal inhibitory concentration (IC_50_).

### 2.10. Statistical Analysis

Data were analyzed statistically according to their distribution, with tests selected based on whether they met the assumptions of normality. When appropriate, results are expressed as mean ± SD or mean ± SEM. Normality was verified using the Shapiro–Wilk test. For datasets with a normal distribution, differences among means were determined by one-way ANOVA followed by Fisher’s least significant difference (LSD) test. In contrast, non-normally distributed data were evaluated using the non-parametric Kruskal–Wallis test, with subsequent pairwise comparisons performed through the Mann–Whitney test. Statistical significance was set at *p* < 0.05. Analyses were carried out with Statgraphics Centurion 18, v18.1.12(Statgraphics Technologies, Inc., The Plains, VA, USA), while Spearman’s rank correlation and forest plot were performed in Python v3.9.4 (Python Software Foundation, Beaverton, OR, USA) within a Google Colab environment.

## 3. Results and Discussion

### 3.1. Changes of Fatty Acids of Dried Murta Berry

From the determination of total lipid content in dried murta samples subjected to FD, LTVD 20, LTVD 30, LTVD 40, and VD 60, the following values were obtained: 4.23, 4.00, 4.24, 4.27, and 4.01% (dry basis), respectively. Therefore, a fatty acid profile analysis was feasible. A total of nine fatty acids were identified and quantified, including four saturated fatty acids (SFAs) and five unsaturated fatty acids (UFAs), with their corresponding contents presented in Table 1.

Among these fatty acids, linoleic acid (69.00–83.26%) and oleic acid (7.02–13.02%) were the predominant UFAs, followed by palmitic acid (an SFA, 5.58–12.75%), each contributing more than 5% to the total fatty acid content. This distribution and relative abundance of fatty acids are in agreement with previous reports on dried murta and its seeds [9,22,26]. As shown in Table 1, individual fatty acids differed significantly (*p* < 0.05) among the drying methods. In comparison to FD, the LTVD 20, LTVD 30, and VD 60 samples showed a marked reduction in palmitic acid and oleic acid, accompanied by an increase in linoleic acid. This redistribution resulted in a higher PUFA/SFA ratio (>9.0) for these methods, whereas FD retained a more balanced ratio (<5.0), which is generally associated with greater oxidative stability relative to the other drying methods [27].

In contrast to conventional atmospheric drying methods, the vacuum-based techniques applied in this study likely minimized lipid oxidation due to low-oxygen conditions. Moreover, the negative pressure environment reduced the water boiling point, thereby limiting thermal stress and helping to preserve the overall fatty acid profile [28]. It should be noted that the fatty acid composition reported here reflects the total fatty acid profile (free and esterified forms combined) obtained after methylation, and therefore does not allow us to distinguish between hydrolyzed and intact lipid fractions.

The observed increase in the PUFA/SFA ratio in LTVD and VD samples may therefore reflect lipid redistribution or the release of PUFAs from complex lipids as a result of structural changes in the cell walls induced by drying, rather than oxidative degradation. In addition, the vacuum conditions are expected to diminish lipid autoxidation, which is typically observed under atmospheric drying when samples reach low water content during the final stages of the process [29]. This interpretation is supported by López et al. [22], who demonstrated that linoleic acid was particularly susceptible to thermal oxidation during atmospheric drying methods such as convective, infrared, and sun drying due to its two double bonds (C=C) [28], as evidenced by its lower levels compared to VD-dried murta. The elevated linoleic acid content observed in dried murta is particularly relevant because this essential fatty acid contributes to prostaglandin synthesis, supports cellular regeneration, and plays a protective role against various cardiovascular disorders [30].

### 3.2. Changes of Tocol Compounds of Dried Murta Berry

Tocols are methylated phenolic compounds, several of which exhibit vitamin E activity and help protect against oxidative stress [31]. Among the tocol compounds, the tocopherols (TPs) in murta after drying were found to consist predominantly of the α-TP isoform, followed by γ-TP, with only minor proportions of β- and δ-TP. Although α-tocotrienol was identified as the primary tocotrienol (TT), it was present in lower amounts, whereas the β- and γ-TT isoforms were detected only in LTVD 30 (Table 2).

The same TP isoforms were also identified by López et al. [22] in dried murta; however, these authors did not report the presence of TTs. Concerning the drying methods used, FD resulted in lower contents of tocols in dried murta, whereas by using LTVD at different temperatures, VD 60 had markedly higher tocol contents compared to FD. Specifically, α-TP content increased by 1616% in LTVD 40, 4419% in LTVD 30, 4906% in VD 60, and 7861% in LTVD 20 (Table 2). As the chemical structure of α-TP contains a chromanol ring and a saturated side chain with multiple methyl groups, it may confer high thermal stability by increasing steric hindrance and reducing the rate of radical addition and polymerization reactions [32]. This feature might help to explain the preservation of TP levels during LTVD (and even in VD 60), where moderate heat did not promote significant degradation. However, this would not explain the reduced TP content observed in FD. Santana et al. [33] speculated that enzymes remaining active during FD might have contributed to the losses of TPs due to the absence of an effective thermal inactivation. Nonetheless, in our study, enzymes are unlikely to be the cause of the low TP content observed in the FD sample, as LTVD also involves mild processing conditions that may not be sufficient to fully inactivate enzymatic activity. We speculate that the lower TP levels detected in FD samples may not necessarily reflect actual degradation, but rather a reduced recovery efficiency during extraction. As FD preserves the structural integrity of cell walls and membranes, the release of lipophilic antioxidants from lipid-rich compartments such as plastids and oil bodies could be limited. In addition, the freezing stage in FD may increase oil viscosity, further hindering the diffusion and extraction of tocols from the cellular matrix. In contrast, LTVD and VD methods may have facilitated the release of TPs from complex lipid matrices through moderate thermal disruption of cell membranes, while applied heat may have reduced oil viscosity, enhancing recovery efficiency [30]. These interpretations remain speculative and should be confirmed in future studies using complementary extraction approaches.

### 3.3. Changes in Bioactive Compounds of Dried Murta Berry

Nine phenolic compounds belonging to three groups were detected and quantified in murta samples subjected to vacuum-based methods, as presented in Table 3.

This table includes (i) four phenolic acids: gallic acid (GA), vanillic acid (VA), ellagic acid (EA), and trans-cinnamic acid (TCA); (ii) three flavonoids: catechin (CAT), epicatechin (EC), and quercetin (Q); and (iii) and two simple phenols: pyrogallol (PYG) and tyrosol (TYR). The identification of nine phenolic compounds has been previously reported in murta fruit, seeds, and pomace [4,6,7,8,9,13]. In addition, the most murta phenolics were found to belong to the flavonoid group [6,8,10,11,34]. Likewise, CAT (46.50 mg/100 g) was the predominant compound, followed by PYG (39.30 mg/100 g) and EC (25.41 mg/100 g), while TCA was not quantifiable in FD-dried murta, which is consistent with previous findings [7]. In the present study, significant differences (*p* < 0.05) were observed in phenolic composition among FD-, LTVD-, and VD-dried samples (Table 3). Among the flavonoids, LTVD- and VD-dried murta exhibited significantly lower levels of CAT but higher levels of EC and Q compared to FD-dried samples. Under vacuum drying conditions, the low-oxygen environment may greatly reduce the likelihood of flavonoid autoxidation; therefore, structural rearrangements such as epimerization are more likely to occur. D’Almeida et al. [35] demonstrated that CAT undergoes epimerization to EC and other derivatives at relatively mild temperatures (starting from 34 °C). This mechanism may explain the decrease in CAT levels and the concomitant increase in EC observed in LTVD and VD samples. In contrast, FD may preserve the integrity of CAT but does not facilitate epimerization or the release of other flavonoids from the cellular matrix due to the absence of thermal input [36]. Regarding phenolic acids, both LTVD and VD significantly increased or maintained the levels of GA, VA, and EA compared with FD. These findings are consistent with previous reports indicating that moderate heat during drying can promote the release of phenolic acids from the plant matrix by disrupting cell structures, thereby converting less soluble or bound forms into more extractable ones [23,37]. Beyond preservation, such processing conditions may also lead to the formation of free-form phenolic compounds through the transformation or partial degradation of more complex precursors, which could contribute to their enhanced bioavailability. This mechanism may also be applied to simple phenols such as PYG and TYR. Nonetheless, the lower PYG levels observed under certain drying conditions may reflect heat-induced polymerization of this highly reactive phenol, which increases its molecular weight and renders it insoluble in conventional extraction solvents [30]. For TYR, such decreases are more likely attributable to volatilization or limited release from the plant matrix, rather than a polymerization episode.

### 3.4. Antioxidant and Anti-Inflammatory Potential of Dried Murta

**Figure 2:**
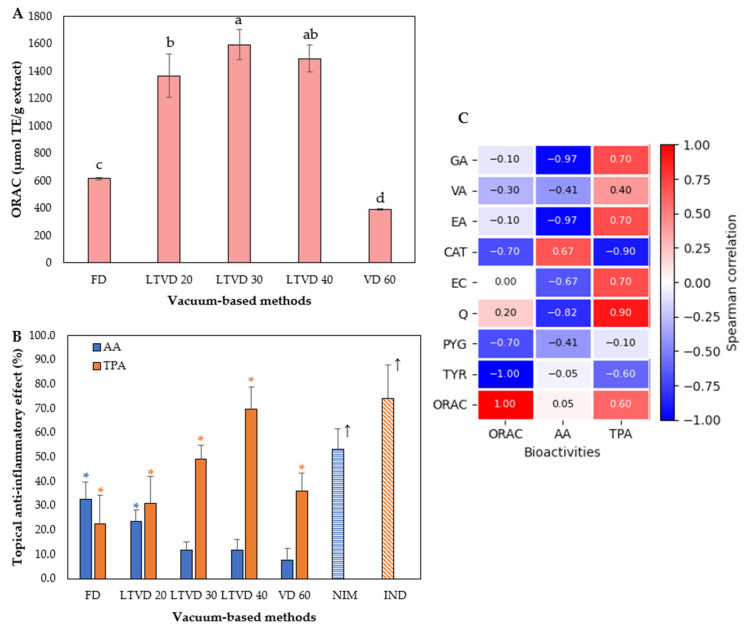
(**A**) Antioxidant potential of murta berry extracts obtained through vacuum-based drying methods (FD, LTVD, and VD), measured by ORAC assays. Data are expressed as mean values of triplicate analyses (*n* = 3), with error bars representing standard deviations. Different letters on bar indicate a statistically significant difference at p < 0.05 (Fisher’s LSD post hoc test). TE: Trolox equivalents. (**B**) Topical anti-inflammatory effects of murta berry extracts, evaluated in a mouse ear edema model induced by arachidonic acid (AA) and phorbol-12-myristate-13-acetate (TPA). Asterisks (blue for AA, orange for TPA) indicate significant differences (* *p* < 0.05) compared with the negative control (100% inflammation) within each model. Nimesulide (NIM) was used as the reference drug for AA, and indomethacin (IND) for TPA. The arrow (↑) indicates the maximum anti-inflammatory effect. Data are expressed as mean ± SEM (*n* = 8 mice per group), with error bars representing SEM. (**C**) Spearman correlation coefficients between individual bioactive compounds and antioxidant and anti-inflammatory effects.

The antioxidant and anti-inflammatory potential of murta extracts obtained from different vacuum-based methods is shown in Figure 2.

Figure 2A shows the antioxidant capacity, expressed as μmol TE/g of extract, as determined by the ORAC assay. The results revealed that the LTVD process preserved the antioxidant capacity more effectively than FD and VD 60. To date, the antioxidant capacity of fresh murta, measured by ORAC, has been previously reported [8,12,38], as well as for dried murta using FD and VD methods [22,23,39]. However, to our knowledge, there are currently no reports on murta being dried using LTVD. Specifically, LTVD 30 showed the highest antioxidant capacity (1592.6 μmol TE/g), followed by LTVD 40 and LTVD 20. These values were more than 2-fold higher than FD (615.7 μmol TE/g) and 4-fold higher than VD 60 (392.9 μmol TE/g). Therefore, our results suggest that LTVD under the tested conditions may enhance the release or retention of antioxidant molecules with high reactivity toward peroxyl radicals. Nonetheless, when we correlated ORAC values with the phenolic compounds identified in this study, weak or even negative correlations were observed (Figure 2C). Flavonoids such as CAT and EC may exhibit limited correlation with ORAC despite their abundance, due to structural features such as intramolecular hydrogen bonding and charge redistribution, which can limit the accessibility and efficiency of the B-ring –OH group in donating hydrogen atoms to peroxyl radicals [40]. Similarly, tyrosol and vanillic acid, both monophenols lacking ortho-dihydroxy structures (catechol sites), typically exhibit higher bond-dissociation energy and lower H-donating capacity, limiting their antioxidant performance in assays based on the hydrogen atom transfer (HAT) mechanism [41]. Furthermore, steric hindrance, poor delocalization, or substitution patterns that reduce H-donating efficiency may also diminish the measurable antioxidant capacity of some phenolic compounds in this ORAC assay [42].

On the other hand, the results of the anti-inflammatory activity, assessed using TPA- and AA-induced ear edema models, are presented in Figure 2B. In both models, the negative control groups developed pronounced edema, which was set as 100% inflammation. As expected, the positive control groups (IND and NIM) exhibited significantly reduced edema compared to the negative controls, with inhibition rates of 74.0% and 53.4%, respectively. Our results showed that topical application of murta extracts significantly reduced ear edema in the TPA model against all treatments (*p* < 0.05). The group treated with the extract obtained from LTVD 40 exhibited an edema reduction of 69.8%, which was the highest among all treatments and comparable to IND. In contrast, the extract obtained from the FD-dried sample was the least active (22.4% edema reduction). On the other hand, in the AA model, only the extracts obtained from FD- and LTVD 20-dried murta exhibited significant inhibitory effects on ear edema (*p* < 0.05), finding that the FD-dried sample extract was the most active (32.7% edema reduction; Figure 2B). The differences between the TPA and AA models can be explained by their distinct inflammatory pathways: TPA upregulates proinflammatory markers [interleukin-1 (IL-1), tumor necrosis factor-alpha (TNF-α), cyclooxygenase-2 (COX-2), inducible nitric oxide synthase (iNOS)], whereas AA activates the COX-2 and 5-lipoxygenase (5-LOX) pathways, leading to prostaglandin and leukotriene synthesis [43,44]. Therefore, antioxidant compounds may also respond differently depending on the pathway. In the TPA-induced ear edema model, reactive oxygen species (ROS) play a pivotal role in amplifying inflammation through activation of nuclear factor kappa B (NF-κB) and subsequent upregulation of proinflammatory mediators such as IL-1, TNF-α, COX-2, and iNOS [45]. The antioxidant capacity of polyphenolic compounds may counteract this cascade by neutralizing ROS, suppressing NF-κB activation, and promoting anti-inflammatory cytokines such as IL-10, thereby mitigating tissue damage [46]. This mechanism may help to explain the strong Spearman correlation observed between phenolic compounds and anti-inflammatory activity in the TPA model (Figure 2C). The strongest correlation (ρ = 0.90) was observed for quercetin (Q), likely due to its extensive number of hydroxyl groups, which play a key role in radical scavenging through hydrogen donation and in metal-chelating activity (e.g., Cu, Fe), ultimately leading to suppressed ROS formation [47]. Alburquenque et al. [39] also suggested that Q present in murta could be one of the compounds involved in the anti-inflammatory effects observed in the native berry, as it has been shown to inhibit NF-κB activation and preserve Nrf2 nuclear translocation under inflammatory conditions.

In contrast, in the AA-induced model, oxidative stress plays a secondary role. Nonetheless, antioxidants may still exert protective effects by limiting lipid peroxidation of arachidonic acid and modulating the NF-κB pathway, which in turn indirectly regulates the COX-2 and 5-LOX expression [44,46]. Therefore, it is not surprising that in this model, only catechin (CAT) showed a positive correlation (*p* = 0.67). This observation may reflect the ability of CAT to interfere with key enzymatic targets in the AA cascade, particularly COX, as previously demonstrated for green tea catechins, which have been shown to significantly reduce eicosanoid production in vivo following ischemic injury [48].

It is important to note that only topical anti-inflammatory effects on skin lesions were evaluated in this study, while dietary effects were not assessed. This distinction is relevant because absorption, distribution, and metabolism of bioactive compounds may differ substantially between dermal and oral routes. In the literature, studies evaluating the anti-inflammatory properties of murta leaf extracts using in vivo topical models—such as TPA- and AA-induced mouse ear edema assays—have consistently reported significant anti-inflammatory activity [3]. Notably, this activity has been attributed not only to flavonoids but also to pentacyclic triterpenoids, including alphitolic, asiatic, ursolic, betulinic, corosolic, madecassic, and maslinic acids [14,15,16]. Future research should therefore focus on investigating the dietary effects of murta berries in order to complement the topical findings and provide a more comprehensive understanding of their potential health benefits.

### 3.5. α-Glucosidase Inhibitory Activity of Dried Murta

**Figure 3:**
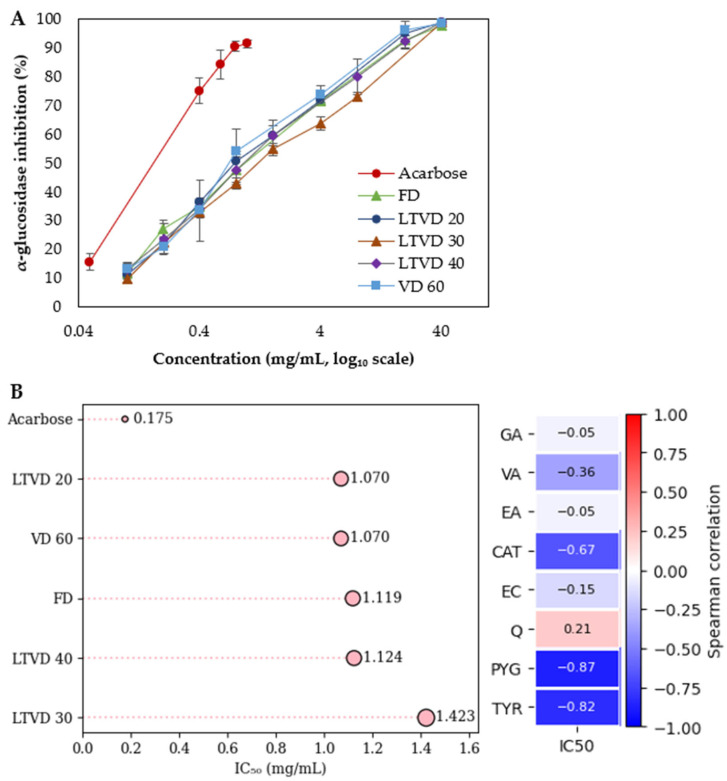
(**A**) α-Glucosidase inhibitory activity at multiple concentrations for acarbose (drug control) and for extracts obtained from murta berries dried using vacuum-based methods (FD, LTVD, and VD). (**B**) Forest plot showing the IC_50_ values for α-glucosidase inhibition by acarbose and murta extracts. The right panel presents Spearman correlation coefficients between individual bioactive compounds and IC_50_ values. Data are expressed as mean values of triplicate analyses (*n* = 3), with error bars representing standard deviations.

α-Glucosidase plays a central role in carbohydrate digestion, and its inhibition can effectively reduce postprandial hyperglycemia; therefore, α-glucosidase inhibitory activity is a key indicator for evaluating the efficacy as a potential inhibitor [13]. Figure 3A presents the comparative α-glucosidase inhibition rates at several concentrations for acarbose (a synthetic inhibitor) and for extracts obtained from dried murta (natural inhibitors) using different vacuum-based drying methods.

The results show that all murta extracts exhibited a clear dose-dependent inhibition of α-glucosidase, with inhibition rates exceeding 90% at the highest tested concentration (~40 mg/mL). At intermediate concentrations (0.8–4 mg/mL), differences among treatments became more evident. LTVD 20 and VD 60 maintained higher inhibition percentages compared to the other drying methods or conditions, particularly in the 0.8–1.6 mg/mL range, suggesting a better preservation of bioactive compounds that could be responsible for α-glucosidase inhibition. FD and LTVD 40 displayed intermediate inhibition, whereas LTVD 30 showed the lowest values in this range. These could be associated with partial degradation or modification of bioactive compounds such as TYR, PYG, and CAT under this LTVD condition (Table 3). At the lowest concentrations tested (<0.4 mg/mL), all extracts showed a marked decrease in inhibition, contrasting with acarbose, which retained over 75% inhibition at 0.4 mg/mL. These concentration–response trends are consistent with the calculated IC_50_ values (Figure 3B). Acarbose exhibited the strongest inhibitory effect (IC_50_ = 0.175 mg/mL), confirming its potency as a positive control. Among murta extracts, LTVD 20 and VD 60 achieved the lowest IC_50_ values (both 1.070 mg/mL), followed closely by FD (1.119 mg/mL) and LTVD 40 (1.124 mg/mL). LTVD 30 presented the highest IC_50_ (1.423 mg/mL), aligning with its lower inhibition percentages at intermediate concentrations. Previous studies have reported significant α-glucosidase inhibitory activity in extracts of murta fruit from different genotypes, with IC_50_ values ranging from 1.4 to 26.0 µg/mL dry extract [8]. Other authors have also demonstrated the inhibitory effects of murta leaf, stem, and fermented juice extracts, with variable IC_50_ values [13,49].

Since a lower IC_50_ value denotes a stronger α-glucosidase inhibition, a negative Spearman correlation indicates that higher concentrations of a compound are associated with greater inhibitory activity. In this study, the inhibitory potential of murta extracts against α-glucosidase appeared to be closely linked to their phenolic composition, particularly compounds such as TYR, PYG, and CAT, which exhibited the most pronounced negative correlations with IC_50_ values (Figure 3B). The strong inhibitory activity of these phenolic compounds can be partly explained by their high hydroxyl group content, which enhances binding affinity to the enzyme through non-covalent interactions such as hydrogen bonding, hydrophobic interactions, and non-covalent interactions between aromatic rings (π–π stacking) [13]. Structural features like ortho-position or multiple substitution hydroxyl groups, seen in compounds such as CAT and PYG, may further improve inhibitory capacity by enabling more hydrogen bonds with amino acid residues in the α-glucosidase active site, as previously observed for other flavonoids with similar substitution patterns [50]. Additionally, anthocyanins present in murta may synergistically enhance inhibition by interacting with substrate-binding regions or inducing conformational changes that enable a reduction in enzymatic activity [49].

### 3.6. Cytotoxic Activity of Dried Murta Against Gastric Adenocarcinoma (AGS) Cells

**Figure 4:**
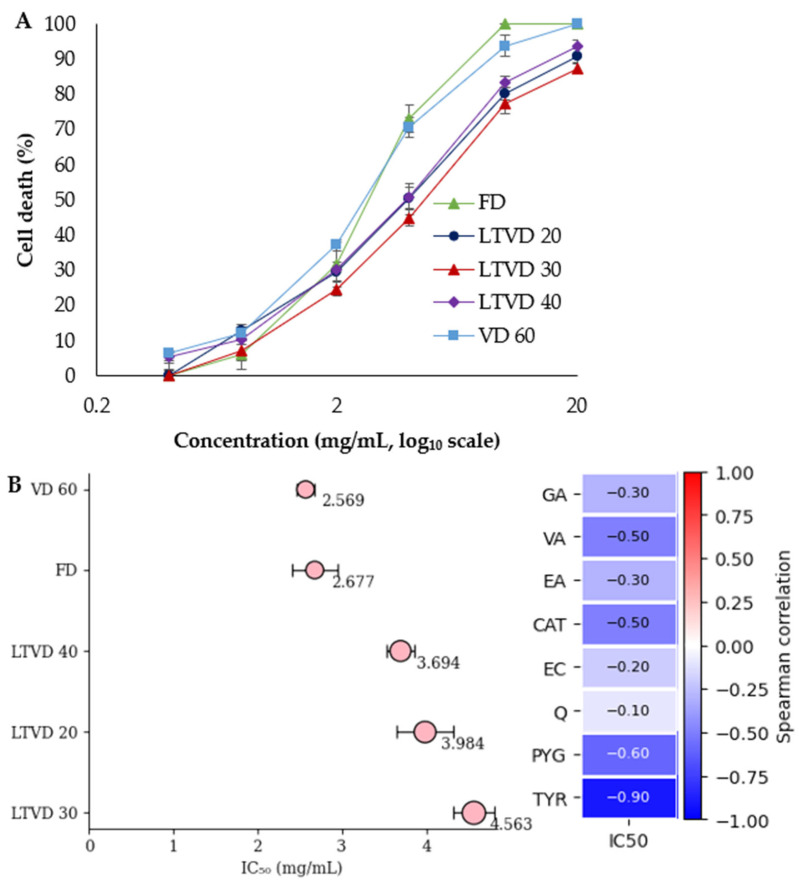
(**A**) Cytotoxic effects at multiple concentrations of murta berry extracts obtained through vacuum-based drying methods (FD, LTVD, and VD) against gastric adenocarcinoma (AGS) cells. (**B**) Forest plot illustrating the IC_50_ values of murta extracts. The right panel presents Spearman correlation coefficients between individual bioactive compounds and IC_50_ values. Data are expressed as mean ± SEM from three independent experiments (*n* = 3), with error bars indicating SEM.

The propidium iodide assay, which detects loss of plasma membrane integrity, was used to evaluate the cytotoxic effect of murta extracts, expressed as a percentage of cell death, against gastric adenocarcinoma (AGS) cells. As shown in Figure 4A, there are clear differences among the dose–response curves of extracts obtained by different vacuum-based drying methods, which were evident in the 0.4–20 mg/mL range.

At 2 mg/mL, the cytotoxicity began to diverge, with VD 60 and FD inducing the highest levels of cell death (37.11 ± 1.63% and 31.47 ± 6.44%, respectively), followed by LTVD 40 (30.31 ± 1.02%) and LTVD 20 (29.62 ± 2.49%), while LTVD 30 showed the lowest effect (24.60 ± 1.94%). This divergence was widened at 4 mg/mL: FD reached 73.00 ± 3.91%, ahead of VD 60 (70.44 ± 2.80%) and LTVD 40 (50.60 ± 2.90%), whereas LTVD 20 and LTVD 30 showed lower cytotoxicity (50.20 ± 4.50% and 44.83 ± 2.36%, respectively). All extracts induced near-complete or complete cell death at 10–20 mg/mL. In contrast, responses at the lowest concentrations (0.4–0.8 mg/mL) were moderate and overlapped within the SEM range (≤ ~13%), indicating minimal distinction among drying methods in this range. These dose–response behaviors closely reflect the IC_50_ values (Figure 4B), where VD 60 and FD exhibited the lowest IC_50_ (2.569 and 2.677 mg/mL, respectively), followed by LTVD 40 (3.694 mg/mL), LTVD 20 (3.984 mg/mL), and LTVD 30 (4.563 mg/mL), establishing a consistent cytotoxic potential ranking: VD 60 ≈ FD > LTVD 40 > LTVD 20 > LTVD 30. Similar comparable findings have been reported in other cancer cell models, where murta extracts obtained through VD and FD exhibited stronger antiproliferative effects against the human NCI-H1975 and mouse HT-22 cell lines, respectively, compared to other drying methods [7].

Spearman’s rank correlations (Figure 4B) among IC_50_ values and phenolic compounds revealed consistently negative associations, indicating that higher concentrations of these bioactive compounds were generally associated with enhanced cytotoxicity (lower IC_50_). Among them, TYR showed the strongest and significant inverse correlation (ρ = −0.90, *p* = 0.037), suggesting that it may play a key role in driving the antiproliferative effects of the extracts. PYG, CAT, and VA also exhibited moderate negative correlations (ρ = −0.60 and −0.50, respectively), whereas GA and EA displayed weaker trends (ρ = −0.30 each). Although these latter associations were not statistically significant, the overall pattern indicates that multiple phenolic compounds act in concert to enhance cytotoxicity. This interpretation aligns with observations in murta leaves, where the whole extract displayed greater cytotoxicity against AGS cells than flavonoid- or tannin-enriched fractions [51], thus reinforcing the notion that synergistic interactions among diverse compounds are essential for inhibiting gastric cancer cell proliferation.

## 4. Conclusions

This study compared LTVD, FD, and VD applied to murta berries. The results showed that LTVD has potential as an alternative to FD and VD for preserving bioactive compounds and maintaining functional properties, although its effects should not be interpreted as outperforming freeze-drying, which is already recognized as a highly effective method for the preservation of bioactive compounds. These findings highlight that different vacuum-based methods influence the chemical composition and bioactivity of murta in distinct ways.

It is noteworthy that the higher levels of some phenolic compounds in LTVD samples may have arisen not only from preservation but also from the release or transformation of bound forms during processing. Furthermore, the study was restricted to a limited number of drying conditions, and the anti-inflammatory evaluation was confined to a topical model of skin lesions, without assessing dietary effects. In addition, mechanistic explanations for the observed bioactivities (anti-inflammatory, α-glucosidase inhibition, and cytotoxicity) were not explored, as they would require more specific molecular and cellular analyses. Future studies should aim to clarify the mechanisms underlying these differences, confirm them across larger datasets, and extend the evaluation to dietary models to better understand the bioavailability and systemic effects of preserved compounds.

From an application perspective, LTVD faces challenges for large-scale adoption, including equipment costs, energy consumption, and throughput. Techno-economic analyses and process optimization will therefore be needed to evaluate the feasibility of LTVD in the valorization of wild murta.

## Figures and Tables

**Table 1:** Quantitative profile of fatty acids in murta berry subjected to vacuum-based methods (FD, LTVD, and VD).

Fatty Acids	Abbreviation	Vacuum-Based Methods				
(%)		FD	LTVD 20	LTVD 30	LTVD 40	VD 60
Palmitic acid	C16:0	12.75 ± 0.21 ^a^	5.58 ± 0.03 ^c^	5.78 ± 0.22 ^c^	6.59 ± 0.18 ^b^	5.80 ± 0.11 ^c^
Stearic acid	C18:0	2.14 ± 0.20 ^a^	2.16 ± 0.00 ^a^	2.02 ± 0.04 ^a^	2.12 ± 0.12 ^a^	2.19 ± 0.11 ^a^
Oleic acid	C18:1n-9	13.02 ± 0.49 ^a^	7.09 ± 0.00 ^c^	7.02 ± 0.01 ^c^	12.23 ± 0.17 ^b^	7.21 ± 0.14 ^c^
Vaccenic acid	C18:1n-7	0.75 ± 0.02 ^a^	0.25 ± 0.00 ^b^	0.23 ± 0.02 ^b^	0.79 ± 0.00 ^a^	0.28 ± 0.07 ^b^
Linoleic acid	C18:2n-6	69.00 ± 0.18 ^c^	82.65 ± 0.10 ^a^	83.26 ± 0.15 ^a^	75.77 ± 0.44 ^b^	83.04 ± 0.20 ^a^
α-Linolenic acid	C18:3n-3	1.77 ± 0.24 ^a^	1.10 ± 0.01 ^b^	1.02 ± 0.02 ^b^	1.93 ± 0.11 ^a^	0.94 ± 0.02 ^b^
Arachidic acid	C20:0	ND	0.53 ± 0.04 ^a^	0.50 ± 0.02 ^bc^	0.58 ± 0.07 ^a^	0.40 ± 0.03 ^c^
Eicosenoic acid	C20:1n-9	ND	0.17 ± 0.00 ^b^	0.16 ± 0.02 ^b^	0.25 ± 0.01 ^a^	0.14 ± 0.01 ^b^
Behenic acid	C22:0	0.56 ± 0.03 ^a^	0.15 ± 0.00 ^b^	ND	ND	ND
SFAs		15.45	8.42	8.30	9.29	8.39
MUFAs		13.77	7.51	7.41	13.27	7.63
PUFAs		70.77	83.75	84.28	77.70	83.98
PUFA/SFA		4.58	9.95	10.15	8.36	10.01

Values are expressed as mean ± standard deviation (*n* = 3). Within each row, values followed by different small superscript letters share significant differences according to Fisher’s LSD post hoc test (at *p* < 0.05). Not detected (ND). SFAs: saturated fatty acids (C16:0, C18:0, C20:0 and C22:0); MUFAs: monounsaturated fatty acids (C18:1n-9, C18:1n-7 and C20:1n-9); PUFAs: polyunsaturated fatty acids (C18:2n-6 and C18:3n-3). Values are expressed as the relative percentage of the fatty acids detected under the applied analytical conditions; minor fatty acids not detected may also be present in murta berries.

**Table 2:** Quantitative profile of tocopherols in murta berry subjected to vacuum-based methods (FD, LTVD, and VD).

Tocopherols	Abbreviation	Vacuum-Based Methods				
(μg/g of Oil)		FD	LTVD 20	LTVD 30	LTVD 40	VD 60
α-Tocopherol	α-TP	7.86 ± 1.14 ^e^	624.61 ± 2.47 ^a^	355.28 ± 4.65 ^c^	134.89 ± 3.71 ^d^	393.37 ± 7.38 ^b^
β-Tocopherol	β-TP	6.11 ± 0.22 ^d^	51.69 ± 2.59 ^b^	64.62 ± 0.49 ^a^	35.33 ± 0.07 ^c^	49.50 ± 0.50 ^b^
γ-Tocopherol	γ-TP	24.99 ± 0.11 ^e^	129.47 ± 0.77 ^b^	170.90 ± 1.25 ^a^	89.79 ± 1.57 ^d^	116.12 ± 2.33 ^c^
δ-Tocopherol	δ-TP	8.21 ± 0.22 ^e^	14.04 ± 0.01 ^d^	38.28 ± 0.16 ^a^	19.39 ± 0.01 ^b^	17.24 ± 0.21 ^c^
α-Tocotrienol	α-TT	7.65 ± 0.21 ^d^	24.42 ± 1.79 ^a^	15.68 ± 0.20 ^b^	8.93 ± 0.89 ^d^	13.47 ± 0.09 ^c^
β-Tocotrienol	β-TT	ND	ND	3.19 ± 0.13 ^a^	ND	ND
γ-Tocotrienol	γ-TT	ND	ND	7.24 ± 0.12 ^a^	ND	ND

Values are expressed as mean ± standard deviation (*n* = 3). Within each row, values followed by different small superscript letters share significant differences according to Fisher’s LSD post hoc test (at *p* < 0.05). Not detected (ND).

**Table 3:** Quantitative profile of phenolic compounds in murta berry subjected to vacuum-based methods (FD, LTVD, and VD).

Phenolic Compounds	Abbreviation	Vacuum-Based Methods				
(mg/100 g of Sample)		FD	LTVD 20	LTVD 30	LTVD 40	VD 60
Gallic acid	GA	1.90 ± 0.19 ^d^	2.06 ± 0.20 ^d^	2.48 ± 0.18 ^c^	3.05 ± 0.09 ^b^	3.74 ± 0.20 ^a^
Vanillic acid	VA	9.48 ± 0.38 ^d^	11.35 ± 0.63 ^c^	9.32 ± 0.45 ^d^	15.95 ± 0.25 ^a^	14.76 ± 0.69 ^b^
Ellagic acid	EA	0.83 ± 0.07 ^d^	1.07 ± 0.01 ^c^	1.23 ± 0.06 ^b^	1.62 ± 0.05 ^a^	1.70 ± 0.09 ^a^
Trans Cinnamic acid	TCA	NQ	NQ	NQ	NQ	NQ
Catechin	CAT	46.50 ± 0.21 ^a^	37.81 ± 0.41 ^b^	12.97 ± 0.28 ^d^	27.91 ± 0.88 ^c^	37.03 ± 0.84 ^b^
Epicatechin	EC	25.41 ± 0.59 ^c^	39.21 ± 1.47 ^b^	38.67 ± 0.38 ^b^	50.23 ± 0.32 ^a^	39.52 ± 0.22 ^b^
Quercetin	Q	2.53 ± 0.05 ^e^	5.24 ± 0.32 ^d^	6.85 ± 0.37 ^c^	10.41 ± 0.17 ^a^	7.34 ± 0.09 ^b^
Pyrogallol	PYG	39.30 ± 1.32 ^d^	59.44 ± 1.87 ^b^	30.34 ± 1.66 ^e^	48.86 ± 0.55 ^c^	66.69 ± 2.31 ^a^
Tyrosol	TYR	10.88 ± 0.71 ^b^	10.19 ± 0.24 ^b^	4.70 ± 0.08 ^d^	9.13 ± 0.27 ^c^	13.55 ± 0.11 ^a^

Values are expressed as mean ± standard deviation (*n* = 3). Within each row, values followed by different small superscript letters share significant differences according to Fisher’s LSD post hoc test (at *p* < 0.05). NQ: Not quantifiable.

## Data Availability

Data supporting the findings of this study are available from the corresponding author upon reasonable request.

## Supplementary Materials

The following supporting information can be downloaded at: https://www.mdpi.com/article/10.3390/antiox14101201/s1, Table S1: Proximate composition analysis in murta berries subjected to vacuum-based methods (FD, LTVD, and VD); Figure S1: Representative chromatogram of phenolic compounds in murta berries dried by vacuum-based methods.
