# Supplementary material for: Exploring the Potential of Low-Temperature Vacuum Drying to Improve the Bioactive Compound Content and Health-Promoting Properties of Chilean Wild Murta
Source: Antioxidants (Basel). 2025 Oct 3;14(10):1201. doi: 10.3390/antiox14101201 (PMC12561544; doi:10.3390/antiox14101201)
Supplement: Supplementary file 1 [file antioxidants-14-01201-s001.zip › antioxidants-3865885-supplementary.pdf]

Table S1: Proximate composition analysis in murta berries subjected to vacuum-based methods (FD, LTVD, and VD).

Parameters	Vacuum-based methods					
(g/100 g d.m.)	Fresh	FD	LTVD 20	LTVD 30	LTVD 40	VD 60
Moisture ǂ	81.71 ± 0.29	6.46 ± 0.11	16.15 ± 0.30	14.34 ± 0.09	12.17 ± 0.03	12.20 ± 0.23
Lipid	0.22 ± 0.10	4.23 ± 0.10	4.00 ± 0.10	4.24 ± 0.18	4.27 ± 0.13	4.01 ± 0.13
Ash	3.54 ± 0.18	3.85 ± 0.28	3.42 ± 0.05	2.98 ± 0.05	3.25 ± 0.10	3.14 ± 0.13
Protein	5.90 ± 0.39	4.98 ± 0.19	5.59 ± 0.21	5.32 ± 0.10	5.36 ± 0.13	5.88 ± 0.12
Crude fiber	12.89 ± 0.42	12.22 ± 0.88	13.13 ± 0.65	13.03 ± 0.01	12.62 ± 0.97	12.43 ± 0.67
a_w *	1.0337 ± 0.0290	0.1320 ± 0.0098	0.6049 ± 0.0018	0.5158 ± 0.0056	0.4335 ± 0.0091	0.4126 ± 0.0011

Values are expressed as mean ± standard deviation (*n* = 3). ǂ values expressed in g/100 g. *dimensionless.

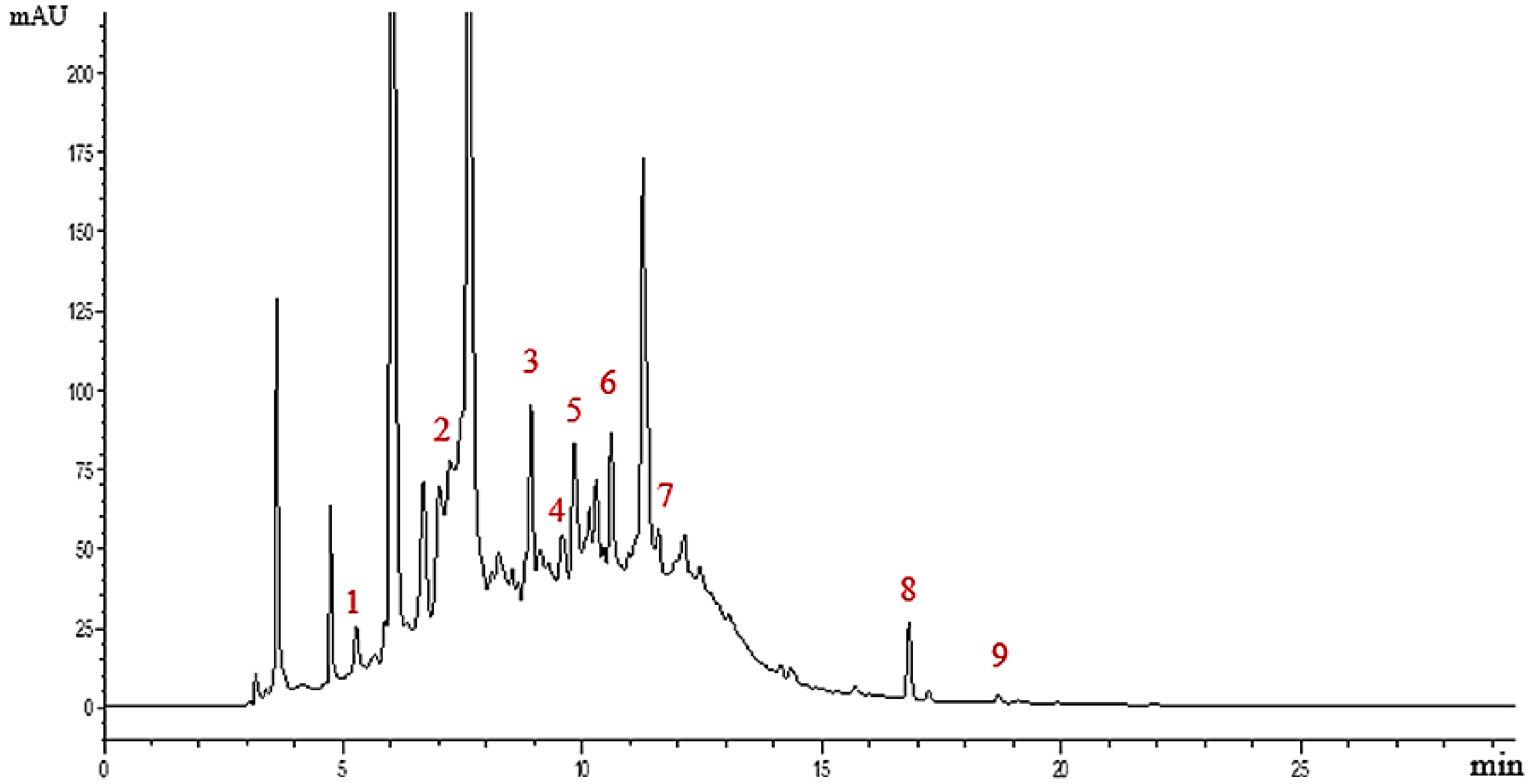

Figure S1: Representative chromatogram of phenolic compounds in murta berries dried by vacuum-based methods. Peaks: 1, Gallic acid; 2, Pyrogallol; 3, Catechin; 4, Tyrosol; 5, Epicatechin; 6, Vanillic acid; 7, Ellagic acid; 8, Quercetin; 9, trans-Cinnamic acid. Detection wavelength: 280 nm.

Supplementary Methods S1. ***In vivo*** **anti-inflammatory assay (AA/TPA)**

The *in vivo* anti-inflammatory experiments were conducted following previously described protocols [14], with minor adaptations, and in compliance with the NIH Guide for the Care and Use of Laboratory Animals and the AVMA 2020 euthanasia guidelines. All procedures were approved by the Institutional Animal Care and Use Committee (CICUA-VID, University of Chile; protocol 25887-ODO-UCH).

- **Animals**

Juvenile BALB/c mice (20–25 g; either sex) were obtained from the Experimental Platform (Animal Facility), Faculty of Dentistry, University of Chile. Mice were used the same day (acute, non-survival study).
Total animals used: 96.

- **Housing and husbandry**

Animals were housed in standard cages (≤4 per cage) with ad libitum access to food and water under controlled macro-environmental conditions until euthanasia.
Diet: LabDiet® JL Rat and Mouse/Auto 6F (code 5K67; ~19.3% protein, 6.2–7.2% fat, 4.3–5.0% crude fiber, ~3.17 kcal/g metabolizable energy); certified/irradiated lots per facility SOPs.
Bedding: Pelleted cellulose bedding (BioFresh™), low-dust and highly absorbent.

- **Experimental groups, randomization, and blinding**

Animals were randomly assigned to groups using simple randomization (shuffled cards). Sex was recorded and approximately balanced (target ≈1:1 when available).
Groups: vehicle (within-animal control), positive reference drugs, and test extracts.
The experimental unit was one mouse.
Personnel performing tissue punching/weighing and statistical analysis were blinded to group allocation (alphanumeric codes).

- **Induction of ear inflammation**

Right ear: arachidonic acid (AA, 2 mg in 20 μL acetone) or phorbol 12-myristate 13-acetate (TPA, 5 μg in 20 μL acetone).
Left ear: 20 μL acetone (within-animal vehicle control).

- **Treatments and positive controls**

Murta (*Ugni molinae*) extracts (solid residue reconstituted in ethanol; 3.0 mg/ear in 20 μL) were applied immediately after the inflammatory stimulus.
Positive controls: nimesulide (NIM; 0.5 mg/ear) for AA, indomethacin (IND; 1.0 mg/ear) for TPA.

- **Euthanasia and tissue collection**

At 1 h (AA) or 6 h (TPA) post-induction, mice were euthanized by CO_2 inhalation (gradual fill). 6-mm ear biopsies were collected from both ears using a sterile mechanical punch.

- **Outcome measures and calculations**

Edema = mass(right ear punch) − mass(left ear punch) per mouse.
Percent inhibition was calculated relative to the median of the stimulus-only group.

- **Sample size determination**

Sample size was determined a priori to achieve 80% power at a two-sided α = 0.05 for the primary outcome, defined as the ear-edema mass difference between the inflamed right ear and the vehicle-treated left ear (OD–OI). Calculations assumed a paired design (each mouse as its own control) with variance estimates from prior datasets in this model, allowing a smaller N while maintaining power. The resulting target was **n = 8 animals per treated group**, with **shared negative controls across sessions** to minimize animal use, as prespecified in the approved CICUA protocol 25887-ODO-UCH. The calculation and allocation plan were performed by a biostatistician (**Dr. Luis Rodríguez**, Instituto de Salud Pública, Chile).

- **Study size & allocation**

Stimulus-only controls: n = 16 per model (AA, TPA).
Treatments and positive controls: n = 8 per treatment or positive control group.
Total animals across both models: n = 96.

- **Inclusion/exclusion criteria and analgesia**

Pre-specified exclusions: pre-existing auricular lesions, clinically relevant distress, or administration errors. No animals met exclusion criteria. Given the acute, same-day endpoint, no anesthetics/analgesics or sedatives were administered to avoid confounding inflammatory pathways; procedures were minimal pain/distress.

- **Welfare monitoring and humane endpoints**

Monitoring at entry to the experimental room, pre-dosing, and every 30–60 min until euthanasia, using a 6-domain ordinal scale (0–3): (i) appearance, (ii) posture/ambulation, (iii) activity/behavior/handling response, (iv) respiration, (v) ear-specific signs, (vi) hydration.
Humane endpoints: any domain = 3, or cumulative ≥4 for ≥10 min ⇒ immediate CO_2 euthanasia; transient 2–3 ⇒ veterinary review and intensified monitoring. No animals reached endpoints.
